# Frailty at ICU admission: a potential alternative to scoring systems based on clinical observation

**DOI:** 10.1007/s11739-025-03976-6

**Published:** 2025-05-24

**Authors:** Yoel Angel, Or Eyal, Dekel Stavi, Nimrod Adi, Yael Lichter, Andrey Nevo, Itay Moshkovits, Daniel Aviram, Idit Matot, Amir Gal Oz

**Affiliations:** 1 Department of Anesthesia, Pain Management and Intensive Care, Tel Aviv Sourasky Medical Center, the School of Medicine, Faculty of Medical and Health Science, Tel Aviv University, Weizmann 6, 6423906 Tel Aviv, Israel; 2 Critical Care Department, University College London Hospital NHS Foundation Trust, London, United Kingdom

**Keywords:** Chronic disease, Clinical frailty score (CFS), Frailty, Geriatrics, Intensive care unit

## Abstract

Frailty is a common clinical syndrome associated with increased risks of morbidity, mortality and other adverse outcomes. It is often used by healthcare providers as a tool for estimating patients` condition and triaging for Intensive Care Unit (ICU) admission, as recommended by several national guidelines. This study aimed to evaluate the utility of observed frailty, a subjective clinical assessment by physicians, as an alternative to the validated Clinical Frailty Score (CFS) and Modified Frailty Index (MFI). Additionally, the study explored the association of these frailty scoring systems with 30 day mortality, mechanical ventilation duration, and ICU length of stay. This retrospective study analyzed 100 patients admitted to an ICU at a tertiary center between March 12 and April 30, 2019. Observed frailty was assessed on a 4-point scale by physicians at ICU admission. CFS (7-point scale) and MFI (11-point scale) were retrospectively calculated. Correlations between these frailty assessments and their relationships with clinical outcomes were examined. Observed frailty correlated moderately with the CFS (Spearman coefficient = 0.4, p < 0.001) but showed a weaker, non-significant correlation with the MFI (Spearman coefficient = 0.18, p = 0.07). Significant differences in 30 day survival were observed based on the 4-point observed frailty scale (p < 0.001). Subjective assessment of frailty using a 4-point scale by physicians at ICU admission correlates with the CFS and is significantly associated with 30 day mortality. This suggests that observed frailty may be a practical tool for clinical decision-making. Further prospective studies are required to validate its utility and prognostic implications.

**Supplementary Information:**

The online version contains supplementary material available at 10.1007/s11739-025-03976-6.

## Introduction

Frailty is a term frequently employed to describe a complex syndrome characterized by the depletion of various reserves, including energy, physical capabilities, cognitive function, and overall health [1–4]. While frailty is a concept widely acknowledged by healthcare professionals as a clinically relevant and valuable construct [5], there is no precise definition for frailty, thus it remains a subject of ongoing exploration. Frailty can be either physical or psychological or a combination of the two [6], and in this study we focus solely on physical frailty.

Several clinical evaluation scales for frailty have been developed. Two widely cited scales are the Clinical Frailty Score (CFS) [5] and the Modified Frailty Index (MFI) [7]. Both scales are related to the Canadian Study of Health and Aging (CSHA) [5] where a measure of frailty was proposed, by counting cumulative clinical deficits from a list of 70 variables—the CSHA Frailty Index.

The CFS is a 7-point scale, with lower scores indicating better physical condition and independence, and higher scores indicating greater frailty and a lack of independence, possibly signifying terminal illness.

In the subsequent NSQIP study [7], 11 criteria from the original 70 within the CSHA Frailty Index were selected for a shorter index: the MFI. That study showed the predictive capability of a score derived exclusively from these selected criteria in assessing the risk of morbidity and mortality among patients following surgical procedures.

Other studies have demonstrated that patients` degree of frailty has prognostic significance: as expected, it was found that frail patients have worse clinical outcomes after surgical interventions [8, 9] as well as after hospitalization in ICUs [10–13] and that frailty can complement other clinical assessment tools such as Sequential Organ Failure Assessment (SOFA) [14] and Acute Physiology and Chronic Health Evaluation-II (APACHE-II) [15, 16].

Guidelines have recommended the use of frailty as a tool to triage patients for ICU admission [17]. In extreme cases, such as during the COVID-19 pandemic, intensive care units faced an extraordinary load and had to prioritize which patient to admit to minimize the loss of life and to ensure that the best possible care is given to those who can best benefit [18]. In such cases, the degree of frailty can be considered during intensive care triage [19], since it can be rapidly assessed, at the bedside, based on information available at the time of presenting to hospital [20].

At our medical center, structured frailty scores are uncommonly used outside the fields of geriatrics and anesthesia, and are rarely used in real-time ICU decision-making, in part due to time constraints and data availability. In contrast, clinicians routinely form a global impression of frailty using available information at bedside. The objective of this study was to estimate whether such an unstructured clinical assessment of patients` degree of frailty, carried out on admission based on routine history and physical examination, is a reliable alternative for the currently accepted frailty scales, and to explore whether it correlates with clinical outcomes.

## Methods

### Study design and population

This was a retrospective cohort study. One-hundred consecutive patients admitted to the ICU in the Tel Aviv Sourasky Medical center between March 12 and April 30, 2019 were included. Patients who were under 18 years of age or who were pregnant were excluded from this cohort. Patients with an ICU admission in the 30 days prior to the index hospitalization were excluded to avoid repeated measurements of the same patient during closely spaced admissions.

Ethics approval and review according to the Declaration of Helsinki [21] for this study were obtained from the hospital’s institutional review board (IRB Approval No. TLVMC-0451-19). As this was a retrospective study, the IRB provided an exemption from obtaining informed consent.

### Data collection

All source data were obtained from the hospital’s medical records. The data were routinely entered into the medical record by the admitting physicians during intake, were available for all participants, and included: sex, age, weight, admission data, past medical history (including cardiovascular risk factors, diagnoses, medications and hospitalizations), pre-hospital functional status (using a 3-point scale: dependent, semi-independent and independent), pre-admission cognitive status. As part of departmental routine on admission, physicians documented a subjective assessment of the patient’s frailty—an “observed frailty score”. This score was an overall impression of the patient, considering all available information at the time of admission, and without the use of any formal scoring system or criteria. A 4-point scale was used—no frailty, mild frailty, moderate frailty or severe frailty.

The CFS [5] and MFI [7] scores, which are measured on 7 and 11-point scales respectively, were retrospectively calculated by one of the authors (OE) for each patient based on review of the medical record set at the time of the initial admission to the ICU.

The original 7-point version of the CFS was used instead of the revised 9-point scale. Furthermore, notwithstanding its original validation in non-ICU patients, the MFI score was included as it was validated on surgical patients, which were anticipated to comprise a substantial portion of the study cohort.

SOFA scores were calculated based on data from the first 24 h following ICU admission. Outcomes at 30 days, including readmissions and mortality, were obtained from medical and administrative hospital records.

Participants were categorized into one of two groups describing the reason for hospitalization—either a “surgical” reason (including postoperative supervision, surgical complications and trauma) or a “medical” (non-surgical hospitalizations).

### Statistical analysis

The sample size was selected to allow for a statistical power (β) of 0.80, assuming an estimated moderate correlation (*r*_*s*_ = 0.3) using the Spearman’s Correlation Test and a significance level (α) of 0.05.

All continuous variables are displayed as means (SD) for normally distributed variables or medians (interquartile range [IQR]) for non-normally distributed variables. Categorical variables are displayed as numbers (%) of participants within each group. Normally distributed continuous variables were compared using a Student’s t-test or Analysis of Variance (ANOVA) tests, non-normally distributed continuous variables using Wilcoxon’s rank sum test and categorical variables using the Chi-squared or Fischer’s exact test. Correlation between different scales was calculated with the Spearman correlation test. Survival analysis was performed using the Log-Rank test. Multivariable logistic regression was used to assess association between different scores and clinical outcomes. First, a univariable analysis was performed on all pertinent variables (Table S2 in the Supplementary Appendix). Only variables that showed a significant association with the outcome on univariable analysis (p < 0.05) were included in the multivariable analysis. A $P$ value of p = 0.05 and below was considered significant.

## Results

### Study population

During the study period, 140 patients were admitted to the hospital’s ICU and their charts were screened for participation in this study. 40 patients were excluded for incomplete data or for not meeting inclusion criteria. Baseline characteristics of the study population are listed in Tables 1 and S1 in the Supplementary Appendix.

**Table 1:** Baseline characteristics of study population (N = 100)

Sex (%)	
Female	40 (40.0)
Male	60 (60.0)
Age, years (median (IQR))	62.5 (40.0–70.0)
Weight, kg (median (IQR))	74.5 (61.5–84.0)
BMI, kg/m^2^ (median (IQR))	25.2 (21.6–27.2)
Pre-hospital functional status (%)	
Independent	83 (83.0)
Semi-dependent	16 (16.0)
Dependent	1 (1.0)
Baseline cognitive decline (%)	4 (4.0)
Any previous admission (%)	55 (55.0)
Admission to ICU directly from ER (%)	27 (27.0)
Pre ICU hospitalization duration, days (mean (SD))	4.2 (6.51)
Smoking (%)	
Current smoker	26 (26.0)
Never smoked	61 (61.0)
Past smoker	13 (13.0)
Admission SOFA score (median [IQR])	4.0 (2.0, 6.0)
Cause for admission—medical (%)	49 (49.0)
Cause for admission—surgical (%)	51 (51.0)
Shock on admission (%)	29 (29.0)
Total number of medications (median [IQR])	4.0 (1.0, 6.2)
Modified frailty index score (median [IQR])	1 (0–2)
Clinical frailty score (median [IQR])	4 (3–5)

*BMI* body mass index (calculated as weight in kilograms divided by height in meters squared), *ICU* intensive care unit, *ER* emergency room, *SOFA* sequential organ failure assessment

Observed frailty assessments were performed by 29 physicians, with each physician contributing a median of 3 assessments (IQR 1–5; Figure S1 in the Supplementary Appendix).

Sixty (60%) of participants were men, and the median age at hospitalization was 62.5 years (IQR 40.00–70.25). Approximately half of the participants (49; 49%) were hospitalized for a reason classified as “medical” and the rest for a reason classified as “surgical”. At the time of admission to the ICU, 17 (17%) of the participants were defined as functionally dependent or semi-dependent, and 4 (4%) had documented cognitive decline. 29 participants (29%) had shock on admission, and the median day-one SOFA index of all participants was 4 (IQR 1.07–1.57).

### Primary outcome: correlation between frailty scores

**Fig. 1:**
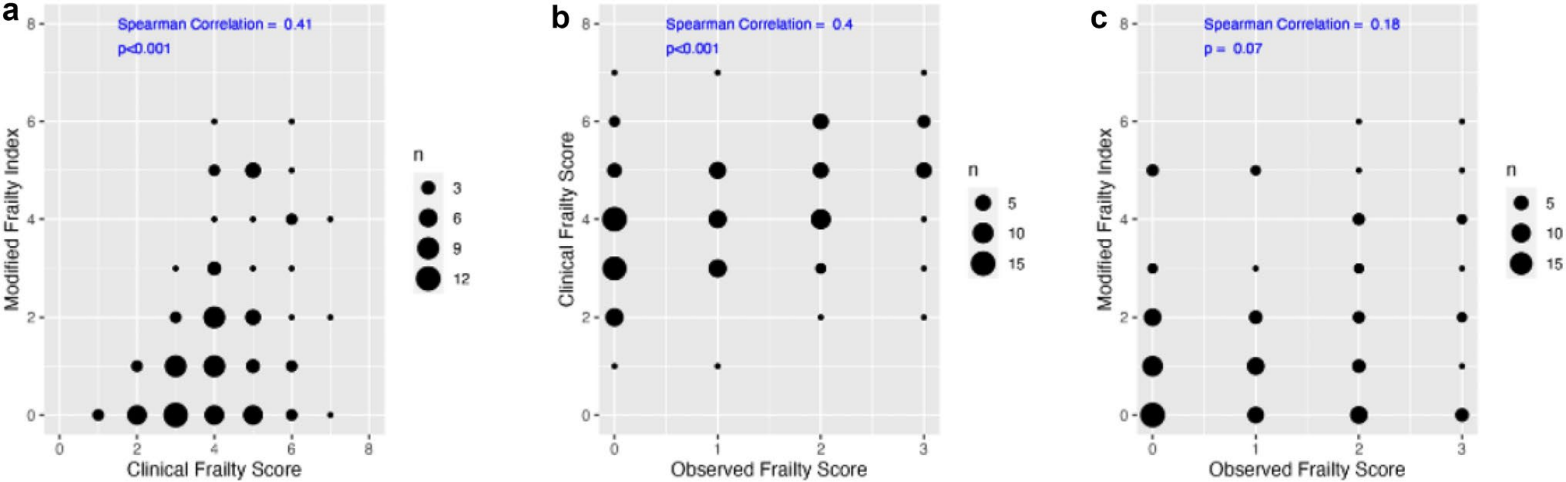
Scatter graphs, the correlation between different frailty scoring systems: the size of the dot on the graph represents the number of patients. **A** Correlation between the two currently accepted frailty scoring systems, clinical frailty score (CFS) and modified frailty index (MFI), calculated retrospectively for each patient. **B** Correlation between the observed frailty assessed by the physician on admission and frailty as assessed retrospectively using the Clinical Frailty Score. **C** Correlation between the observed frailty assessed by the physician and frailty as assessed retrospectively using the Modified Frailty Index

A moderate yet statistically significant correlation was found between the CFS and MFI scores (Spearman coefficient = 0.41, p < 0.001; Fig. 1A) and between the observed frailty assessed by the admitting physician and the frailty scores calculated on the CFS scale (Spearman coefficient = 0.4, p < 0.001; Fig. 1B). The correlation between the observed frailty and MFI scores was weaker and did not reach statistical significance (Spearman coefficient = 0.18; p = 0.07; Fig. 1C).

A post-hoc power analysis based on the observed correlation (rₛ = 0.4) between observed frailty and CFS indicated a statistical power of 98.7% (α = 0.05), suggesting the sample size was adequate to detect a moderate correlation.

### Secondary outcome: association of the observed frailty score with clinical outcomes

**Fig. 2:**
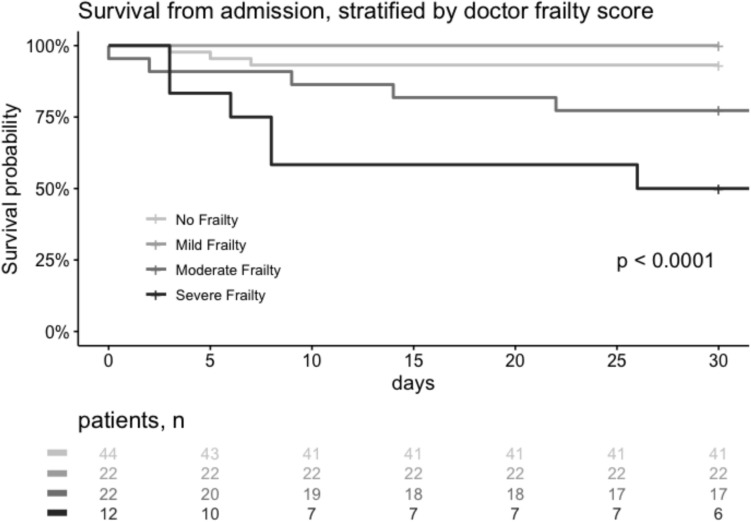
Kaplan-Mayer curve of 30 day mortality, stratified by the observed frailty score

Observed frailty, as assessed by medical staff on admission, was associated with significant differences in 30 day survival (p < 0.001; Fig. 2). On multivariable logistic regression, only day-1 SOFA scores and observed frailty as assessed by the medical staff were associated with survival 30 days after admission, while CFS and MFI were not. The results of the multivariable analysis are presented in Table 2; univariable results are detailed in Table S2 in the Supplementary Appendix.

**Table 2:** Multivariable analysis, 30 days survival

	OR	95% C.I	
		Lower	Upper
Age	1.00	0.96	1.05
Female	0.95	0.16	5.49
Pre-hospital functional status—semi-dependent	1.23	0.16	9.28
Pre-hospital functional status—dependent	0.00	0.00	Inf
Day-one SOFA	1.30	1.07	1.57
Observed frailty score by physician	2.16	1.01	4.61

The McFadden’s pseudo-R2 for the model is 0.342

*SOFA* sequential organ failure assessment

The median duration of ICU stay was 3 days (IQR 2–5). Among the 48 patients who required mechanical ventilation, the median duration of mechanical ventilation was 3 days (IQR 2–7). No significant differences were noted according to the observed frailty score (Table S3 in the Supplementary Appendix).

## Discussion

This study examined whether “observed frailty” can be a valid alternative to accepted frailty scoring systems by examining the correlation between assessment of observed frailty and two currently accepted scores, as well as the ability to predict 3 clinical outcomes—30 day mortality, duration of ICU stay and the number of mechanical ventilation days.

A moderate correlation (*r*_*s*_ = 0.4) was observed between the observed frailty score and the Clinical Frailty Score, while the correlation of observed frailty with the Modified Frailty Index was weaker (*r*_*s*_ = 0.18) and did not reach statistical significance.

Further, a significant association was observed between the observed frailty score and 30 day mortality, but not with duration of ICU stay or of mechanical ventilation.

These results are consistent with previous research in the field that demonstrated an association between frailty and mortality [22]. A meta-analysis [23] examined the relationship between frailty, measured among other factors by CFS and MFI, and clinical outcomes in intensive care, also revealed that frailty was associated with increased risk of hospital and long-term mortality, but not with duration of ICU stay or mechanical ventilation, in line with the findings of this study.

Moreover, in our study, other functional measures such as pre-hospital activity level and the SOFA score [14] were also significantly associated with 30 day mortality. Conversely, The CFS and MFI scores were not significantly associated with this clinical outcome. These findings may be attributed to differences in the patient population under investigation compared to those on which the MFI and CFS scores were originally validated.

Patients hospitalized in ICU are of different ages and suffer from various medical conditions, usually acute in onset and without prior functional deficits. These factors differentiate the population in this study from the populations examined in previous studies, which included a relatively homogeneous population: The NSQIP study that introduced the MFI score exclusively included patients undergoing surgical procedures and were under 50 years of age, whereas our study encompassed both surgical and medical patients with a wider age distribution; the CSHA-FI study [5], which first introduced the CFS score, also included only patients above the age of 65 while the median age in this study was 62.5 years (IQR 40–70). This may have impacted the perceived frailty scores as frailty may be less expected or less clearly expressed in younger adults.

While subjective assessment of frailty is less reproducible than an assessment based on pre-defined scores, it allows for a rapid evaluation of a patient’s pre-morbid condition, which can be particularly useful when triaging admissions to the ICU. The results of this study suggest that such a subjective assessment may have prognostic value and could reasonably be considered as an additional criterion—alongside other clinical factors—when making ICU admission decisions. Given the differences in patient characteristics—particularly age distribution and baseline functional status—compared to the original CFS and MFI validation cohorts, it should be emphasized that the results of this study should not be interpreted as demonstrating superiority. Nonetheless, observed frailty may serve as a practical alternative or complement to structured frailty scores, especially in situations where time or data availability is limited.

*Limitations*: this study has several limitations that should be considered. First, this was a single center, retrospective study which may limit the generalizability of its findings. Second, in the population examined in this study, not many participants had poor functional status upon admission and 44% of patients were assessed as having “no frailty”. This may indicate the presence of a selection bias. While this is a basic characteristic of the population hospitalized in the ICU at this institution, it may also limit the generalizability of the findings to other patient populations. Third, while both the MFI and CFS scores have been previously validated, it is plausible their use in the setting of this study was associated with lower predictive values, because they were calculated retrospectively. However, the fact that a moderate correlation (*r*_*s*_ = 0.41) was observed between both these scores is an indicator of validity. Moreover, calculating these scores retrospectively enabled a blinded structure, avoiding bias from exposing caregivers (who assessed observed frailty) to an accepted frailty score. Yet, bias may still have been introduced, as a staff member’s estimation of frailty may have influenced their treatment decisions, for example withholding aggressive treatment from a patient perceived to be too frail to receive it. Such an effect may have later translated to an “artificial” observed association between perceived frailty and outcomes.

Lastly, while our sample was adequately powered for the primary outcome, the multiple analyses performed for secondary outcome analysis introduce a risk of type I errors, while the limited sample size may have reduced our ability to detect weaker associations in secondary outcomes, increasing the potential for Type II errors.

## Conclusions

A subjective assessment of frailty on a four-point scale, by physicians upon admission to the ICU, was found to be associated with both the CFS score and with 30 day mortality. Further prospective studies are needed to validate these results and make observed frailty more commonplace.

## Supplementary Information

Below is the link to the electronic supplementary material.

Supplementary file1 (DOCX 215 KB)

## Data Availability

The datasets generated during and/or analyzed during the current study are available from the corresponding author upon reasonable request and subject to national and institutional policies.

## Declarations

### Clinical trial registration

This study does not involve a clinical trial; therefore, registration is not applicable.
