# Supplementary material for: Frailty at ICU admission: a potential alternative to scoring systems based on clinical observation
Source: Intern Emerg Med. 2025 May 24;21(2):665–71. doi: 10.1007/s11739-025-03976-6 (PMC13061817; doi:10.1007/s11739-025-03976-6)

**Supplementary Appendix**

**Frailty at ICU Admission: A Potential Alternative to Scoring Systems Based on Clinical Observation**

Yoel Angel, MD, MBA ; Or Eyal, MD; Dekel Stavi, MD; Nimrod Adi, MD; Yael Lichter, MD; Andrey Nevo, MD; Itay Moshkovits, MD, PhD; Daniel Aviram, MD; Idit Matot, MD; and Amir Gal Oz, MD, MBA

**Table S1: Additional baseline characteristics of the study population (N=100)**

| Hypertension | 30 (30.0) |
| --- | --- |
| Diabetes Mellitus | 25 (25.0) |
| Chronic Obstructive Pulmonary Disease (COPD) | 10 (10.0) |
| Previous Myocardial Infarction | 14 (14.0) |
| Peripheral Vascular Disease (PVD) | 7 (7.0) |
| Congestive Heart Failure (CHF) | 20 (20.0) |
| Previous ischemic stroke of transient ischemic event (TIA) | 9 (9.0) |
| Known kidney disease | 14 (14.0) |
| Chronic dialysis status | 3 (3.0) |
| Active Leukemia or Lymphoma in previous year | 2 (2.0) |
| Active solid malignancy in previous year | 23 (23.0) |
| Number of chronic medications prescribed (median [IQR]) | 4.00 [1.00, 6.25] |
| Use of antiaggregants | 32 (32.0) |
| Use of anticoagulants | 13 (13.0) |
| Use of Beta Blockers | 29 (29.0) |
| Use of Statins | 26 (26.0) |
| Use of ACE inhibitors or ARBs | 20 (20.0) |
| Use of diuretics | 16 (16.0) |
| Use of Insulin | 12 (12.0) |
| Use of other oral diabetes medication | 12 (12.0) |

*ACE = Angiotensin Converting Enzyme; ARB = Angiotensin Receptor Blocker.*

**Table S2: Variables included in univariable analysis:**

| Sex |
| --- |
| Age |
| Smoking |
| Baseline Cognitive Status |
| Baseline Functional Status |
| Number of previous hospital admissions |
| Admission reason (medical/surgical) |
| Presence of shock on admission |
| Sequential Organ Failure Assessment (SOFA) score on admission |
| Modified Frailty Index (MFI) |
| Clinical Frailty Score (CFS) |
| Observed Frailty Score |

**Table S3: Differences in duration of ICU stay and mechanical ventilation
according to observed frailty**

|  | **No  Frailty** | **Mild  Frailty** | **Moderate Frailty** | **Severe  Frailty** | **p** |
| --- | --- | --- | --- | --- | --- |
| **Duration of ICU Stay, Days (median [IQR])** | 4.0 [2.0, 6.0] | 3.00 [2.0, 5.0] | 3.0 [2.2, 5.0] | 4.5 [2.0, 6.0] | 0.666 |
| **Duration of Mechanical Ventilation, Days (median [IQR])** | 3.0 [2.0, 7.5] | 2.0 [1.0, 4.0] | 3.0 [2.0, 8.0] | 5.0 [4.0, 6.7] | 0.336 |

Estimation of mechanical ventilation was done only for the subset of patients that were ventilated one or more days (n=48).

**Figure S1: Number of patients seen by each of the 29 physicians.**X axis shows each of the 29 physicians (names redacted).
Blue bars, aligned with the left vertical axis, show number of patients estimated by each physicians.
Red trend line, aligned with the right vertical axis, shows a cumulative percent of patients assessed by each additional physician.


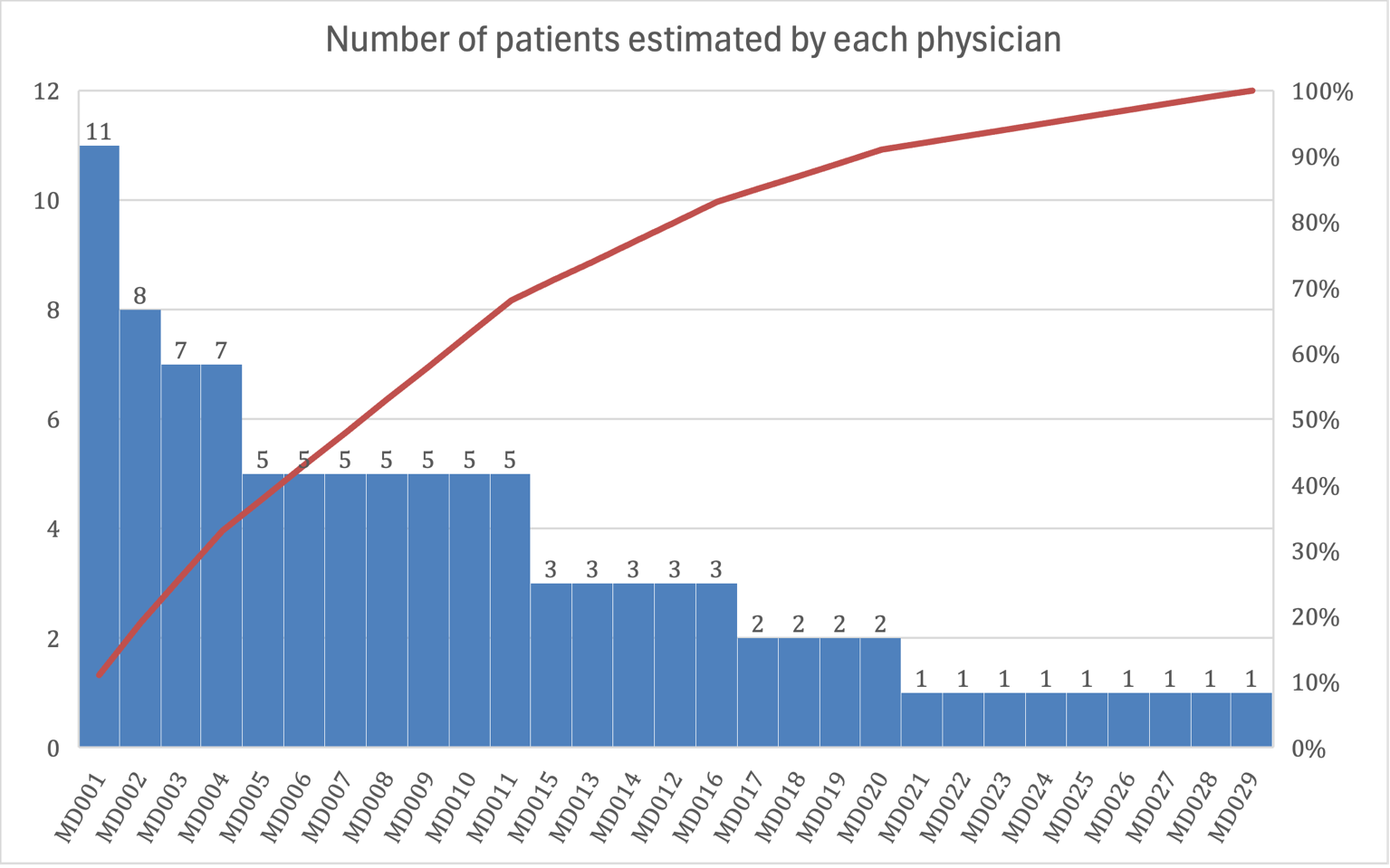

Supplement: Supplementary file 1 — Supplementary file1 (DOCX 215 KB) [file 11739_2025_3976_MOESM1_ESM.docx]
